# Profile of the Intervention Potential of the Phylum Actinobacteria Toward Quorum Sensing and Other Microbial Virulence Strategies

**DOI:** 10.3389/fmicb.2019.02073

**Published:** 2019-10-04

**Authors:** Hema Bhagavathi Sarveswari, Adline Princy Solomon

**Affiliations:** Quorum Sensing Laboratory, Centre for Research in Infectious Diseases (CRID), School of Chemical and Biotechnology, SASTRA Deemed to be University, Thanjavur, India

**Keywords:** anti-biofilm, anti-virulence, microbial natural product, Actinobacteria, anti-pathogenic agents

## Abstract

The rapid dissemination of antimicrobial resistance amongst microorganisms and their deleterious effect on public health has propelled the exploration of alternative interventions that target microbial virulence rather than viability. In several microorganisms, the expression of virulence factors is controlled by quorum sensing systems. A comprehensive understanding into microbial quorum sensing systems, virulence strategies and pathogenesis has exposed potential targets whose attenuation may alleviate infectious diseases. Such virulence attenuating natural products sourced from the different phyla of bacteria from diverse ecosystems have been identified. In this review, we discuss chemical entities derived from the phylum Actinobacteria that have demonstrated the potential to inhibit microbial biofilms, enzymes, and other virulence factors both *in vivo* and *in vitro.* We also review Actinobacteria-derived compounds that can degrade quorum sensing signal molecules, and the genes encoding such molecules. As many Actinobacteria-derived compounds have been translated into pharmaceutically important agents including antibiotics, the identification of virulence attenuating compounds from this phylum exemplifies their significance as a prospective source for anti-virulent drugs.

## Introduction

Antimicrobials have remained the only mode of prophylaxis and therapeutics for microbial infections since its discovery. In the past century, antimicrobials have undeniably revolutionized clinical practices, laying the foundation for breakthroughs in surgeries, organs transplantations, cancer therapy, treatment of burns and trauma wounds, subsequently improving human health. However, the current antimicrobial resistance (AMR) era threatens the reversal of all breakthroughs achieved thus far (Brown and Wright, 2016; Marston et al., 2016). In the United States alone, AMR contributes to 2 million infections and 23,000 deaths per year, substantially increasing the medical expenses by up to 20 billion US dollars each year (Gelband et al., 2015; Centers for Disease Control, and Prevention, 2017). Healthcare agencies across the world have prioritized AMR, and the scientific community has proposed and developed many innovative strategies including the discovery of novel drug targets and other alternative therapeutic interventions to minimize the development of antimicrobial resistance amongst pathogens (Marston et al., 2016).

Virulence factors produced by pathogens are constructive in deteriorating host fitness during infection. A virulence factor could be a structure, or a product, or a strategy that enables the pathogen to gain access and survive within the non-colonized region or cellular compartment of the host. Adhesins, enzymes (invasins and internalins), toxins (hemolytic, cytolytic and neurotoxins), and superantigens are some crucial virulence factors expressed by a pathogen to damage the host’s physiological condition (Hill, 2012). In several pathogens, the expression of these virulence factors are regulated by a cell density-dependent signaling system called quorum sensing (QS) system (Miller and Bassler, 2001; Fetzner, 2015). QS system enables microorganisms across inter and intraspecies within a community to initiate controlled and co-ordinated behavior (Greenberg, 2003; Kaufmann et al., 2008). Although many facets of the intricate prokaryotic QS system remain undeciphered, the available knowledge on the domain’s diverse QS systems provides many targets for the development of drugs that could inhibit the expression of virulence. Given the unrelatedness of virulence to the viability of a pathogen, the cultivation of resistance toward the anti-virulence agent through selective pressure is presumed to be non-existential (Clatworthy et al., 2007).

What is so paramount in the evolution of bacteria is the co-development of secondary metabolites that can disrupt the QS signal molecules and attenuate the virulence of other microorganisms. The competency to disrupt the QS signal molecules [quorum quenching (QQ)] could have been evolved in quorum sensing bacteria to remove or repurpose its own QS signal molecules, or the signal molecules of microorganisms that co-inhabit a competitive environment (Grandclément et al., 2016). Bacteria could have also evolved molecules for degrading N-acyl homoserine lactone (AHL) to utilize AHL as a sole source of carbon and nitrogen, or as armor against antibiotic-producing bacteria (Gonzalez and Keshavan, 2006).

Since the discovery of the AHL degrading enzyme AhlD (acyl homoserine lactone degradation enzyme) from *Arthrobacter* sp. IBN 110 (Park et al., 2003), and the demonstration of the attenuation of *Erwinia carotovora* pathogenesis in transgenic plants expressing autoinducer inactivating aiiA gene from *Bacillus* sp. (Dong et al., 2001), an array of bacterial natural components with quorum quenching properties have been reported. These include secondary metabolites produced by bacteria from various phyla including Actinobacteria, Bacteroidetes, Firmicutes, Proteobacteria, and Cyanobacteria. In this article, we review the natural compounds from the phylum Actinobacteria that have been reported to produce AHL degrading enzymes, the corresponding genes, and other Actinobacteria derived compounds that inhibits or attenuates microbial virulence both *in vitro* and *in vivo* (Tables 1, 2).

**TABLE 1:** Compounds identified from Actinobacteria displaying anti-virulence properties.

**Serial No.**	**Organism**	**Source**	**Protein/Compound/Enzyme**	**Target/Reporter Organism**	**Anti-virulence activity**	**References**
**Family: *Micrococcaceae*; Genus: *Arthrobacter***						
1	*Arthrobacter* sp. Arth410	Beach mud and the homogenates from fishes and seaweeds	Dextranase (Dex410)	*Streptococcus mutans* ATCC 25175	Inhibition of biofilm formation	Jiao et al., 2014
2	*Arthrobacter* sp. B4	Soil	B4-EPS1	*Pseudomonas aeruginosa* PAO1	Inhibition of biofilm formation	Li et al., 2015
3	*Arthrobacter* sp. PGVB1	Sandstone	Arthroamide Turnagainolide A	*Staphylococcus aureus* agr reporter strain 8325-4	Reduces the luminescent level of the agr-dependent gene expression	Igarashi et al., 2015
4	*Arthrobacter oxydans* KQ11-1	Marine	Dextranase	*Streptococcus mutans*	Inhibition of adhesion and biofilm formation	Wang et al., 2016
**Family: *Brevibacteriaceae*; Genus: B*revibacterium***						
5	*Brevibacterium casei MSA19*	*Dendrilla nigra*	Glycolipid surfactant	Clinical isolates of human and fish pathogens and marine biofilm forming bacteria	Inhibit biofilm formation	Kiran et al., 2010
6	*Brevibacterium casei* MS104	*Dendrilla nigra*	Poly-hydroxy butyrate (PHB) polymer	*V. vulnificus V. fischeri V. parahaemolyticus V. alginolyticus V. harveyi*	Inhibition of adhesion	Kiran et al., 2014
7	*Brevibacterium casei* MS104	*Dendrilla nigra*	Poly-hydroxy butyrate (PHB) polymer	*Vibrio campbelli* strain PUGSK8	Inhibition of motility, hemolysis activity and biofilm formation	Kiran et al., 2016
**Family: *Nocardioidaceae*; Genus: *Kribbella***						
8	*Kribbella* sp. BFI 1562	Soil	Protease	*Staphylococcus aureus* ATCC 25923 *Staphylococcus aureus* ATCC 6538	Inhibition of Biofilm formation Dispersion of preformed biofilms	Park et al., 2012
**Family: *Nocardiopsaceae*; Genus: *Nocardiopsis***						
9	*Nocardiopsis dassonvillei subsp. dassonvillei* XG-8-1	Marine sediments collected from the seashore	Nocapyrone H Nocapyrone I Nocapyrone M	QS reporter strains; *P. aeruginosa* QSIS-lasI25 and *Chromobacterium violaceum* CV026	Inhibition of QS controlled gene expression	Fu et al., 2013
10	*Nocardiopsis* sp. GRG1 (KT23540)	Brown algae	Zinc oxide nanosheets	Multi Drug Resistant *Proteus mirabilis* BDUMS1 and *Escherichia coli* BDUMS3	Inhibition of biofilm and attenuate biofilm architecture	Rajivgandhi et al., 2018
**Family: *Streptomycetaceae*; Genus: *Streptomyces***						
11	*Streptomyces* sp. TOHO-Y209 *Streptomyces* sp. TOHO-O348	Soil	Piericidin A1, 3′-rhamnopiericidin A1, Piericidin E	*Chromobacterium violaceum* CV026	Inhibition of violacein production	Ooka et al., 2013
12	*Streptomyces* sp. NIO 10068	Marine Invertebrate	Cinnamic acid Linear dipeptide (Pro-Gly and N-amido- α- proline)	*Pseudomonas aeruginosa* ATCC 27853	Reduction of motility, formation of biofilm, production of pyocyanin, production of rhamnolipid, production of Las A protease, swimming and twitching	Naik et al., 2013
13	*Streptomyces* sp. *BFI 250*	Soil	Protease	*Staphylococcus aureus* ATCC 25923 *Staphylococcus aureus* ATCC 6538	Inhibition of Biofilm formation Dispersion of preformed biofilms	Park et al., 2012
14	*Streptomyces parvulus*	*Codonopsis lanceolata*	Actinomycin D	MSSA ATCC 25923 MSSA ATCC 6538 MRSA ATCC 33591	Reduce formation of biofilm, hemolysis, slime production and hydrophobicity of bacterial cell.	Lee et al., 2016
15	*Streptomyces parvulus HY026*	Sea water	Actinomycin D	*Pseudomonas aeruginosa* PAO1 *Staphylococcus aureus* 95005	Reduction in biofilm formation	Miao et al., 2017
16	*Streptomyces fradiae* PE7	Estuarine sediment	Quercetin	*Anabaena* species and *Nostoc* species	Reduction of germination of spores	Gopikrishnan et al., 2016
17	*Streptomyces coelicoflavus* S17	Soil	1H-pyrrole-2-carboxylic acid Docosanoic acid	*Pseudomonas aeruginosa* PAO1 *Pseudomonas aeruginosa* PAO1	Reduction in the production of elastase, protease and pyocyanin. Elimination of expression of las genes and rhl/pqs cascade. Reduction in the production of elastase, protease and pyocyanin.	Hassan et al., 2016
18	*Streptomyces* sp. CCB-PSK207	Marine sediment	Fatty acid methyl esters	*C. elegans* infected with *Pseudomonas aeruginosa* PA14	Induction of host immunity	Fatin et al., 2017
19	*Streptomyces* sp. OUCMDZ-3436	*Enteromorpha prolifera*	4-Hydroxy-3-methyl-6-propylpyridin-2(1H)-one 3-Ethyl-4-hydroxy-6-isopropylpyridin-2(1H)-one 4-Hydroxy-6-isobutyl-3-methylpyridin-2(1H)-one (S)-6-(sec-Butyl)-4-hydroxy-3-methylpyridin-2(1H)-one	*Pseudomonas aeruginosa* ATCC10145	Inhibition of gene expression controlled by quorum sensing in *Pseudomonas aeruginosa* QSIS-lasI.	Du et al., 2018
20	*Streptomyces albus*/pAlnuori*Δaln6*	Recombinant Strain	Alnumycin D	*Staphylococcus aureus* ATCC 25923	Inhibition of biofilm	Oja et al., 2015
21	*Streptomyces violaceoruber* Tü22	Microbial culture collection	Granaticin B	*Staphylococcus aureus* ATCC 25923	Inhibition of biofilm	Oja et al., 2015
22	*Streptomyces minutiscleroticus* M10A62	Mangesite mine soil	Selenium nanoparticle (SeNP)	Antibiotic resistant *Acinetobacter* species	Inhibition of biofilm	Ramya et al., 2015
23	*Streptomyces* sp. *SCSGAA 0027*	*Subergorgia suberosa*	Hygrocin C	*Bacillus amyloliquefaciens* SCSGAB0082	Inhibition of biofilm, adhesion, EPS production, cell motility, and surface hydrophobicity	Wang et al., 2018
24	*Streptomyces* sp. *CNQ343*	Seafloor sediment sample	Bahamaolide A	*C. albicans* ATCC 10231	Inhibition of Isocitrate lyase (ICL) in glyoxylate cycle	Lee et al., 2014
25	*Streptomyces xanthocidicus KPP01532*	Natural product library	Piericidin A and Glucopiericidin A	*Erwinia carotovora subsp. atroseptica* (Eca) *C. violaceum* CV026	Reduction of soft rot disease symptoms in potato Inhibition of violacein production	Kang et al., 2016
26	*Streptomyces* sp. strain MC11024	Library of culture extracts of actinomycetes	Streptorubin B	MRSA N315	Inhibition of biofilm formation	Suzuki et al., 2015
27	*Streptomyces* sp. ZL-24	Wet soil	Melanin	*P. aeruginosa* ATCC 9027 and *S. aureus* ATCC 6538	Inhibition of biofilm formation	Wang et al., 2019
28	*Streptomyces* sp. AT37	Desert soil	5-[(5E,7E,11E)-2,10-dihydroxy-9,11-dimethyl-5,7,11-tridecatrien-1-yl]-2-hydroxy-2-(1-hydro-xyethyl)-4-me- thyl-3(2H)-furanone	Methicillin sensitive *Staphylococcus aureus* and methicillin resistant *Staphylococcus aureus*	Inhibition of biofilm	Driche et al., 2017
29	*Streptomyces griseoincarnatus* HK12	*Callyspongia* sp.	9Z-Octadecenal, arachidic acid, erucic acid, 13Z-Octadecenal and tetracosanoic acid.	*Pseudomonas aeruginosa* and *Staphylococcus aureus*	Inhibition of biofilm	Kamarudheen and Rao, 2019
30	*Streptomyces* Strain K01-0509	Soil	Guadinomines A and Guadinomines B	Enteropathogenic *E. coli*	Inhibition of type III secretion system	Iwatsuki et al., 2008
31	*Streptomyces* sp. *ANK313*	soil	Khatmiamycin Aloesaponarin II	Zoospores of *Plasmopara viticola*	Inhibition of motility	Abdalla et al., 2011
32	*Streptomyces* TOHO-M025	Soil	Maniwamycins	*C. violaceum* CV026	Inhibition of violacein production	Fukumoto et al., 2016
33	*Streptomyces* sp. *MC025*	Unidentified red alga	Collismycin C	Methicillin sensitive *Staphylococcus aureus* and methicillin resistant *Staphylococcus aureus*	Inhibition of biofilm	Lee et al., 2017
34	*Streptomyces* sp. *strain FA-70*	Soil	FA-70C1 (Phenylalanyl-ureido-cit-rullinyl-valinyl-cycloarginal)	*Porphyromonas gingivalis* ATCC33277 and *Porphyromonas gingivalis* KDP129	Inhibition of Arg-gingipain (Rgp)	Kadowaki et al., 2003
35	*Streptomyces griseorubens* AU2	Soil	Silver nano particles	*Pseudomonas aeruginosa* ATCC 27853 and *Staphylococcus aureus* ATCC 25923	Inhibition of biofilm	Baygar and Ugur, 2017
36	*Streptomyces* sp. OUCMDZ-3436	*Enteromorpha prolifera*	1. 4-hydroxy- 3-methyl-6-propylpyridin-2(1H)-one 2. 3-ethyl-4-hydroxy- 6-isopropylpyridin-2(1H)-one 3. 4-hydroxy-6-isobutyl-3-meth- ylpyridin-2(1H)-one 4. (S)-6-(sec-butyl)-4-hydroxy-3- methylpyridin-2(1H)-one	*Pseudomonas aeruginosa* QSIS-lasI biosensors	Inhibition of QS controlled gene expression	Du et al., 2018
**Others**						
37	*Actinomycete* strain DSW812	Soil, Marine sediment, Sea water and Plants	WS9326A and WS9326B	VirSR system of *Clostridium perfringens S. aureus 8325–4 (type-I AIP), K12 (type-II AIP) and K9 (type-IV AIP) S. aureus Newman and S. aureus K3*	Suppression of expression of pfoA (Perfringolysin O) Inhibition of the production of hemolysin Reduction of S. aureus cytotoxicity in human corneal epithelial cells	Desouky et al., 2015

**TABLE 2:** Extract from Actinobacteria displaying anti-virulence activity.

**Serial No.**	**Organism**	**Origin**	**Medium**	**Target Organism**	**Activity**	**Concentration**	**References**
1	*Actinomycetes* C5-5Y	Library collection	Partially purified yellow pigment	*Staphylococcus aureus and Streptococcus mutans*	Inhibition of biofilm formation, inhibition of protease and lipase activity, reduction in cell surface hydrophobicity	10 μg/ml	Soundari et al., 2014
2	*Kribbella* sp. BFI 1562	Library collection	Spent medium	*Pseudomonas aeruginosa*	Inhibition of biofilm formation	1% (v/v)	Kim et al., 2012
3	*Streptomyces albus* A66	Marine Sediment	Extract	*Vibrio harveyi*	Inhibition of biofilm formation and dispersal of mature biofilms.	2.5% (v/v)	You et al., 2007
4	*Streptomyces akiyoshiensis* CAA-3	Coral reef	Methanolic extracts	*S. aureus* ATCC 11632, methicillin resistant *S. aureus* ATCC 33591	Biofilm inhibition Inhibition of intestinal colonization in *C. elegans*	0.1 mg/ml	Bakkiyaraj and Pandian, 2010
5	*Streptomyces akiyoshinensis A3 Actinobacterium* sp. A10	*Acropora digitifera*	Ethyl acetate extract	*Streptococcus pyogenes*	Inhibition of Biofilms	10–200 μg/ml	Nithyanand et al., 2010
6	*Streptomyces* sp. A745	Arctic Sediment	Diethyl ether fraction	*Vibrio cholerae* (MCV09)	Inhibition of Biofilm	200 μg/ml	Augustine et al., 2012
7	*Streptomyces* sp. BFI 230	Actinomycetes culture library	Spent medium	*Pseudomonas aeruginosa*	Inhibition of biofilm formation and Interference in iron uptake	1% (v/v)	Kim et al., 2012
8	*Streptomyces* sdLi	Marine sediment	Ethyl acetate extract	*Proteus mirabilis* UCB4	Suppression of urease production and swarming motility Inhibition of biofilm	10 mg/ml 15 mg/ml	Younis et al., 2016
9	*Streptomyces* sp. SBT343	*Petrosia ficiformis*	Ethyl acetate extract	*Staphylococcus epidermidis*, methicillin resistant *Staphylococcus aureus* and methicillin sensitive *Staphylococcus aureus*	Inhibition of biofilm	62.5–250 μg/ml	Balasubramanian et al., 2017
10	*Streptomyces albogriseolus* GIS39Ama	Soil	Ethyl acetate extract	(1) *Klebsiella pneumoniae* MTCC 3384, (2) *Vibrio cholerae* MTCC 3906, (3) *Escherichia coli* MTCC 687 and (4) *Pseudomonas aeruginosa* MTCC 2453	Inhibition of biofilm Reduction in EPS production, biofilm density and viability	1. 625 ppm 2. 625 ppm 3. 312 ppm 4. 1250 ppm	Lokegaonkar and Nabar, 2017
11	*Nocardiopsis* sp. ZoA1	*Zingiber Officinale*	Spent medium	Multidrug-resistant *Staphylococci capitis* 267 and *Staphylococci haemolyticus* 41	Inhibition of biofilm	200 μg/ml	Sabu et al., 2017
12	*Nocardiopsis* sp. A731	Arctic Sediment	Culture supernatant	*Vibrio cholerae* (MCV09)	Inhibition of Biofilm	20% (v/v)	Augustine et al., 2012

## Family: *Micrococcaceae*; Genus: *Arthrobacter*

*Arthrobacter* was one of the first genera in the phylum Actinobacteria reported to possess a gene dedicated to the degradation of AHL. *Arthrobacter* sp. IBN110 demonstrated the potential to degrade AHLs of different lengths and acyl side chains including N-3-oxohexanoyl-L-homoserine lactone (OHHL), N-octanoyl-L-homoserine lactone (OHL), and N-3-oxododecanoyl-L-homoserine lactone (OdDHL) (Park et al., 2003). When OHHL producing *Erwinia carotovora N98* was co-cultured with strain IBN110, the concentration of OHHL and OHHL mediated pectate lyase activity significantly reduced, indicating the potential of IBN110 to disrupt AHL. Indeed, the strain IBN110 possessed acyl homoserine lactone degradation gene (ahlD) that encoded AhlD protein with characteristic zinc-binding motif HXDH≈H≈D crucial for N-acyl homoserine lactonase (AHLase) activity (Dong et al., 2002). HPLC and mass spectrometry analysis revealed that AhlD hydrolyzed the ester bond in N-acyl homoserine lactone molecules and released the homoserine lactone ring. Multiple sequence alignment of AhlD with the other known AHLases, including AttM and AiiA revealed < 26% overall sequence similarity (Park et al., 2003).

*Arthrobacter* sp. PGVB1 derived arthroamide and turnagainolide A (cyclic depsipeptides) demonstrated the ability to inhibit agr signaling in a *Staphylococcus aureus* agr reporter strain. At 5–10 μM concentrations, the compounds suppressed the expression of the agr-dependent gene without cytotoxicity. The inhibitory concentration value (IC_50_) of arthroamide and turnagainolide A against *Staphylococcus aureus* agr reporter strain was 0.3 and 0.8 μM, respectively (Igarashi et al., 2015). The *Arthrobacter* B4-EPS1 exopolysaccharide abolished *Pseudomonas aeruginosa* biofilms at a lower concentration (about 86.1% at 50 μg/mL) than the exopolysaccharides reported from other genera (Li et al., 2015) including EPS (exopolysaccharide) from *Streptococcus phocae* PI80 (about 20% inhibition at 1 mg/mL) (Kanmani et al., 2011), r-EPS (released-exopolysaccharide) from *Lactobacillus acidophilus* A4 (about 80% inhibition at 1 mg/mL) (Kim et al., 2009), and A101 from *Vibrio* sp. QY101 (about 75% inhibition at 100 μg/mL) (Jiang et al., 2011). The B4-EPS1 exopolysaccharide also expressed broad-spectrum inhibitory activity against the *Staphylococcus epidermidis, Enterococcus faecium, Klebsiella pneumonia, Escherichia coli, and Morganella morganii* biofilms (Li et al., 2015). Dex410, a dextranase from marine *Arthrobacter* sp. strain (Arth410) inhibited biofilms of *Streptococcus mutans* with minimum biofilm inhibitory concentration (MBIC_50_) ranging between 1.27 and 6.35 μM/ml. Dex410 also reduced the 24 h biofilms of *Streptococcus mutans* with minimum biofilm reduction value (MBRC_50_) of 3.81–8.89 μM/ml. This concentration was significantly lesser than the antibacterial chlorhexidine (MBRC_50_ > 20 μM) present in the commercially available oral care products. The animal experiment showed that long term usage of Dex410 effectively prevented dental caries (Jiao et al., 2014). *Arthrobacter oxydans* KQ11-1 derived dextranase displayed MBIC_50_ and MBIC_90_ values of 2 U/m1 and 6 U/ml, respectively toward *Streptococcus mutans* biofilm. The MBRC_50_ against preformed *Streptococcus mutans* biofilm was 5 U/ml and the dextranase decreased the thickness of the biofilm up to 36.67 μm (Wang et al., 2016).

## Family: *Brevibacteriaceae*; Genus: *Brevibacterium*

In 1959, when Grecz and his team reported the inhibitory activity of culture filtrates of *Brevibacterium linens* ATCC 9174 and *Brevibacterium linens* ATCC 9175 toward the germination of *Clostridium botulinum* type A spores, little did they know that it was one of the earliest reports of anti-infective property ever reported from the Genus *Brevibacterium* (Grecz et al., 1959). In fact, it was only during the mid 2000s that the evidence of quorum sensing in *Clostridium botulinum* and its role in regulating the germination of botulinum spores was established (Zhao et al., 2006). Today, out of the 51 known species of *Brevibacterium*^1^ only two strains from *Brevibacterium casei* (*Brevibacterium casei* MSA19 and MS104), both interestingly isolated from the marine sponge *Dendrilla nigra*, have been reported to produce compounds with anti-virulence property against bacterial pathogens (Kiran et al., 2010, 2016; Table 1).

At a concentration of 30 μg/ml, *Brevibacterium casei* MSA19 glycolipid affected the formation of biofilm by inhibiting the initial attachment of the bacteria mediated by pili and flagella. At a very low concentration, the *Brevibacterium* glycolipid significantly reduced the formation of both individual and mixed bacterial biofilms (Kiran et al., 2010; Table 1). Microtiter plate assay and CLSM images revealed that polyhydroxy butyrate (PHB) derived from *Brevibacterium casei* MSI04 suppressed the adhesion of pathogenic *Vibrio* species on both polystyrene and glass surfaces at a concentration of 0.6 mg (200 μl). In fact, the PHB was most effective in inhibiting the formation of biofilm than dislodging pre-formed biofilm (Kiran et al., 2014). At 50 μg/ml concentration, PHB inhibited bioluminescence, and at 150 μg/ml reduced the formation of *Vibrio campbellii* PUGSK8 biofilm. Infection of *Vibrio* species in brine shrimp (*Artemia* sp.) is typically fatal, and, treatment of ≥ 50 μg/ml of PHB resulted in the elicitation of protection to shrimps up to 48 h. This research revealed that the ß-hydroxy butyric acid, an intermediate released during the PHB degradation indeed regulates the expression of the virulence factors in PUGSK8 (Kiran et al., 2016).

## Family: *Mycobacteriaceae*; Genus: *Mycobacterium*

The discovery of AHL lactonases in *Mycobacterium* was an outcome of exploration for the establishment of promiscuity of the divergence of bacterial phosphotriesterase (PTE), an enzyme first discovered in *Pseudomonas diminuta* with efficient paraoxonase activity (Raushel and Holden, 2000; Roodveldt and Tawfik, 2005). The absence of naturally occurring specific substrate and the evolutionary elusiveness of PTE led to a BLAST search for genes homologs to *Pseudomonas diminuta* PTE. Three genes including two from the phylum Actinobacteria; PPH (putative parathion hydrolase) in *Mycobacterium tuberculosis* and AhlA (N-acyl-homoserine lactone acylase) in *Rhodococcus erythropolis* sharing a 34 and 28% identity and SsoPox (phosphotriesterase with natural lactonase activity) from an archeon *Sulfolobus solfataricus* with 31% identity were identified (Afriat et al., 2006). The PPH and AhlA have been classified as phosphotriesterase-like lactonase (PLL) from the amidohydrolase superfamily that hydrolysis substrates with either ester or amide functional groups at phosphorus and carbon centers (Seibert and Raushel, 2005). A subsequent exploration into the enzymology of PPH and AhlA revealed that the paraoxonases activity was rather a promiscuous function that could have emerged in PLLs from its progenitor lactonase activity (Afriat et al., 2006).

Expression of PPH gene in *Escherichia coli* in the presence of three metal ions (Zn^2+^, Co^2+^ and Mn^2+^) prompted a 2000-fold increase in PPH’s lactonase activity than the paraoxonase activity. Further research revealed that these metal ions were vital for PPH’s enzymatic activity and that metal chelation inactivated PPH. The K_*M*_ and k_*cat*_/K_*M*_ values of PPH during the hydrolyzes of lactones ranged between e20 and 230 μM, and from 1.4 × 10^4^ to 5 × 10^5^ s^–1^ M^–1^, respectively. The k_*cat*_/K_*M*_ values generally increased with six membered lactone ring and lactones with longer and more hydrophobic side chains. However, no visible lactonase activity against N-acyl thiolactone analog derived from homocysteine was observed (Afriat et al., 2006). Another orthologous of PLL, MCP (AHL lactonase from *Mycobacterium avium* subsp. *paratuberculosis* K-10), also degraded a wide range of AHLs and displayed up to 92% sequence similarity with PPH. MCP also demonstrated low paraoxonase activity indicating that the naturally occurring substrate for MCP does not contain phosphate esters. Introduction of a single point mutation in ßα loop at the carboxyl-terminal end of eighth β-strand of the MCP resulted in a mutant (N266Y) with enhanced AHL lactonase activity than the wild type MCP. The N266Y mutant (substitution of TAC for AAC at 266 codon) increased the k_*cat*_/K_*M*_ values up to 4 to 32-fold for C12-HSL and C6-HSL than the wild type. Further research with the mutants including the N266 showed that a suitable amino acid substitution at the 266 residue, and its proximity to the lactone ring of AHL provide the possibility to enhance AHL lactonase activity by introducing an AHL binding geometry (Chow et al., 2009).

## Family: *Microbacteriaceae*; Genus: *Microbacterium*

Several strains of *Microbacterium* species isolated from potato tuber plant (*Solanum tuberosum*) have been reported to degrade AHLs with both short and long acyl side chains (Morohoshi et al., 2009; Wang et al., 2010, 2012). An infestation of *Pectobacterium carotovorum* subsp. *carotovorum* in potato crop results in soft rot disease, a consequence of coordinated expression of virulence factors mediated by QS signal molecule N-(3-oxohexanoyl)-L-homoserine lactone (Chatterjee et al., 1995). Two endophytic strains: *Microbacterium testaceum* StLB018 and *Microbacterium testaceum* StLB037 attenuated virulence in *Pectobacterium carotovorum* subsp. *carotovorum* NBRC 3830 without bactericidal activity (Morohoshi et al., 2009). Nucleotide sequence analysis of StLB037 revealed a complete open reading frame encoding a protein of 295 amino acids that belonged to α/ßhydrolase fold family encompassing the characteristic catalytic active site Gly-X-Ser-X-Gly (Holmquist, 2000). Named as autoinducer inactivation gene from *Microbacterium testaceum* (aiiM), the expression of StLB037 AiiM protein in the NBRC 3830, drastically reduced the pectinase production and also attenuated tissue maceration non-bactericidally (Morohoshi et al., 2009). HPLC analysis with fraction containing maltose binding protein-AiiM (MBP-AiiM) fusion protein and C10-HSL produced two peaks that coordinated with the standards of C10-HSL, and the opened lactone ring of C10-HSL. As this established the role of AiiM in degrading AHL, further study revealed that AiiM was not influenced by the length or the substitution of the acyl side chains. The partially purified MBP-AiiM protein exhibited relatively better activity against C12-HSL and 3-oxo-substituted AHLs than C6-HSL, C8-HSL, C10-HSL and other unsubstituted AHLs (Wang et al., 2010).

Investigation into the distribution and diversity of AiiM among the Genus *Microbacterium* with various strains isolated from different sources including potato plant, scarlet runner bean, rapeseed, Chinese paddy, milk, cheese, air, soil, activated sludge, imperial moth and many more, exposed that the superior level of AHL degradation exhibited by the *Microbacterium* strains was due to the presence of aiiM gene encoded in the chromosome of bacterium. Out of 26 *Microbacterium* strains included in the study, only 9 strains exhibited high degrading ability against C6-HSL, 3OC6-HSL, C10-HSL, and 3OC10-HSL. Remarkably, these strains were of potato plant origin and were positive for aiiM gene in their genetic material. The remaining 17 strains lacked the ability to degrade C6-HSL and exhibited low to relatively intermediate degrading ability against 3OC6-HSL, C10-HSL, and 3OC10-HSL. These strains were of non-potato origin and were negative for aiiM gene in their DNA (Wang et al., 2012). Comparison of the nine aiiM positive strains with phylogenetically related *Microbacterium* strains (*Microbacterium* sp. *PcRB024* and *M. testaceum ATCC 15829*) revealed the absence of significant AHL degrading activity or the aiiM gene in the chromosome. The aforementioned evidence led to a conclusion that the aiiM was not conserved among the Genus *Microbacterium* and could have spread amongst the *Microbacterium* strains inhabiting potato tuber ecosystem through the non-horizontal mode of transmission supposedly due to the absence of transposons flaking the aiiM (Wang et al., 2012). Although *Microbacterium testaceum* aiiM homologous gene with high sequence similarities have been identified in other actinobacterial strains including *Rhodococcus erythropolis* PR4 and *Rhodococcus opacus* B-4, their expression as MBP-AiiM protein lacked AHL lactonase activity. The *Microbacterium* StLB037 encoded AiiM bears < 15% similarity with other known AHL lactonases including AidP, AiiA, AttM, AhlD, QsdA, QlcA, BpiB01, BpiB04 and BpiB07. The absence of conserved zinc-binding domains found in AHL lactonases from metallo-ß-lactamase super family and PTE family proteins affirmed the novelty and ingenuity of AiiM (Wang et al., 2010).

## Family: *Nocardiopsaceae*; Genus: *Nocardiopsis*

The culture supernatant of cold temperature adapted *Nocardiopsis* sp. A731, at a concentration of 20% (v/v) inhibited about 80% of *V. cholerae* biofilm (Augustine et al., 2012). Three novel α-pyrones; nocapyrone H **(1)**, nocapyrone I **(2)**, and nocapyrone M **(3)** (Tables 1, 3), were extracted from *Nocardiopsis dassonvillei* subsp. dassonvillei XG-8-1 inhibited QS controlled virulence in *P. aeruginosa* QSIS-lasI biosensor and *Chromobacterium violaceum* CV026 at a concentration of 100 μg/mL (Fu et al., 2013). At 200 μg/ml concentration, the crude extract of *Nocardiopsis* sp. ZoA1 inhibited the formation of *Staphylococcus haemolyticus* 41 and *Staphylococcus capitis* 267 biofilms by ≥ 90% (Table 2). Dose-dependent biofilm inhibition assay with ZoA1extract supported the assumption that inhibition of multidrug-resistant coagulase negative staphylococci (CONS) was due to the inhibition of production of proteinaceous factors and exopolysaccharide. However, ZoA1 strain also possessed broad-spectrum antibacterial activity against *Staphylococcus aureus, Bacillus subtilis, Salmonella typhi*, and *Vibrio cholerae* (Sabu et al., 2017). The spent medium of soil *Nocardiopsis* sp. TRM 46200 showed ≥ 90% inhibition against the *Staphylococcus epidermidis* biofilms for over 24 h. The major metabolite in the culture supernatant was proteinous in nature and exhibited both antibiofilm and protease activity. The crude protein derived from TRM 46200 reduced the cell surface hydrophobicity, and also degrade DNA and the extracellular polymeric substance (EPS) of *Staphylococcus epidermidis* strains (ATCC 35984 and 5-121-2) (Xie et al., 2018). The culture supernatant of *Nocardiopsis* sp. GKU 213 inhibited biofilm formation of *Staphylococcus aureus* ATCC 25923 by 60% without anti-bacterial activity (Leetanasaksakul and Thamchaipenet, 2018). Zinc oxide nano-sheets (ZnO NSs) produced by *Nocardiopsis* sp. GRG1 (KT23540) effectively inhibited the biofilms of multi-drug resistant *Proteus mirabilis* BDUMS1 and *Escherichia coli* BDUMS3 by 92 and 90%, at 20 μg/ml concentration, respectively. CLSM images and fluorescent light microscopic analysis showed that ZnO NSs disintegrated the biofilm architecture of uropathogens, by dispersing the bacterial cells leaving only fewer adherent cells and cell aggregates (Rajivgandhi et al., 2018).

**TABLE 3:** Metabolites derived from Actinobacteria exhibiting virulence inhibitory activity.

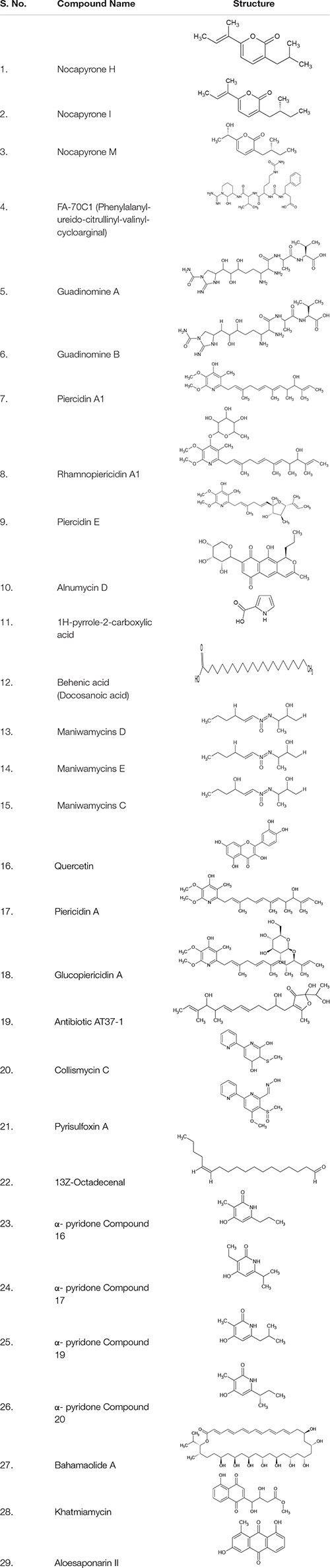

## Family: *Nocardiceae*; Genus: *Rhodococcus*

While the possible presence of γ-butyrolactone dependent quorum sensing system in *Rhodococcus* species could be understood only by *in silico* genomic analysis of *Rhodococcus erythropolis* PR4 and *Rhodococcus* strain RHA1, the quorum quenching mechanism of this genera is one of the well-established among bacteria (Wuster and Babu, 2007; Latour et al., 2013). Indeed, *Rhodococcus* sp. is a unique organism possessing three different mechanisms for N-acyl homoserine lactone degradation; an AHL lactonase, an oxidoreductase and an amidase (Uroz et al., 2003, 2005, 2008, 2009; Park et al., 2006), unraveling the unprecedented evolution of multiple QQ strategies within a bacterium.

In 2003, Uroz and his team demonstrated that a ‘wild type’ *Rhodococcus erythropolis* W2 can degrade C6-HSL, and attenuate the QS-regulated pathogenesis in *Pectobacterium carotovorum subsp. carotovorum*, a pathogen of potato tubers, without limiting or inhibiting its growth (Uroz et al., 2003). Although primarily identified on the basis of its ability to utilize 3-oxo-C6 HSL, the *Rhodococcus erythropolis* W2 interestingly degraded the 3-oxo derivative of acyl homoserine lactone less efficiently than the other known AHL degrading bacteria (Leadbetter and Greenberg, 2000). The broad substrate specificity, rapid AHL inactivation and interference with QS regulated pathogenesis exhibited by *Rhodococcus erythropolis* W2, instigated a series of studies to understand the underlying catabolic mechanism involved in AHL degradation (Uroz et al., 2003, 2005, 2009). Incubation of N-(3-oxooctanoyl)-L-homoserine lactone (3O,C8-HSL), N-(3-oxodecanoyl)-L-homoserine lactone (3O,C10-HSL), N-(3-oxododecanoyl)-L-homoserine lactone (3O,C12-HSL), N-(3-oxotetradecanoyl)-L-homoserine lactone (3O,C14-HSL) with whole cells of W2 in phosphate buffer saline resulted in the production of 3-hydroxy derivatives: 3OH,C8-HSL, 3OH,C10-HSL, 3OH,C12-HSL and 3OH,C14-HSL, respectively. This reaction was mediated by oxidoreductase activity (Uroz et al., 2005). The broad substrate specificity of oxidoreductase also catalyzed the reduction of AHL derivatives substituent with aromatic acyl side chains or without lactone ring including N-(3-oxo-6-phenylhexanoyl) homoserine lactone and 3-oxododecanamide, respectively (Uroz et al., 2005).

Interestingly, the oxidoreductase activity observed in the whole cell of *Rhodococcus erythropolis* W2 was absent in the culture extract. The complete elimination of unsubstituted and substituted (3-oxo or 3-hydroxy) AHLs from the incubation medium containing the culture extract of W2, suggested the presence of another mechanism to degrade AHL. This was later validated to be an acylase that catalyzed AHL degradation by releasing dansylated homoserine lactone from the incubated reaction mixture of N-(3-oxodecanoyl)-L-homoserine lactone and cell culture extract of W2 strain. The AHL acylase cleaved the amide bond of both short and long chain AHLs yielding homoserine lactone through amidolytic activity (Uroz et al., 2005).

Identification of a soil bacterium that displayed the potential to utilize AHL led to the discovery of AHL lactonase, the third mechanism for the catabolism of AHL in *Rhodococcus* species (Park et al., 2006). Two strains of *Rhodococcus* sp. LS31 and PI33 displayed different substrate specificity for N-3-oxo-hexanoyl-L-homoserine lactone (OHHL), and mass spectrometric analysis revealed that both the strains hydrolyzed the lactone ring of AHL (Park et al., 2006). *Rhodococcus* sp. strain LS31 degraded AHL of different lengths with different acyl side chain substitutions, contradicting the higher degrading activity exhibited by *Rhodococcus erythropolis* W2 against 3-oxo-substituent AHLs than unsubstituted AHLs (Uroz et al., 2003, 2005). The AHL lactonase from both the strains LS31 and PI33 destroyed AHL, while the *R. erythropolis* W2 attenuated the signal molecules (Park et al., 2006). Although much of the enzymology underlying *Rhodococcus* AHL acylase and AHL oxidoreductase has been unraveled, the genetic determinant of these enzymes still remains unknown.

QsdA, a product of the gene qsdA (quorum sensing signal degradation), was reported as the another AHL lactonase utilized by *Rhodococcus erythropolis* strain W2 to degrade AHL. This novel class of AHL lactonase did not show homology to any previously reported AHL degrading enzymes that were characterized from the two protein super families: Zinc-dependent glyoxylase and N-AHSL amidohydrolases of the β lactam acylases (Uroz et al., 2008). In fact, the QsdA belonged to the group of phosphotriesterase (PTE) like lactonase (PLL) within the amidohydrolase superfamily (Hawwa et al., 2009) that possessed the characteristic binuclear metal center inside a TIM- barrel (β/α)_δ_ - barrel-shaped scaffold). Though initially this enzyme was described as paraoxonases due to their activity against organophosphate pesticide paraoxon (Afriat et al., 2006), later experiments showed that the enzymes also hydrolyzed lactones including the N-acyl homoserine lactones with 6 to 14 carbon in acyl side chains, irrespective of carbon 3 substitution (Uroz et al., 2008). The qsdA operon can also be utilized for the assimilation of various lactone in the milieu including the γ- lactone, and also for the disruption of QS signals of competitive bacteria (Latour et al., 2013). The qsdA homologue is conserved in reference strains including, *Rhodococcus erythropolis* DCL14 (de Carvalho and da Fonseca, 2005) and it was suggested that the detection of AHL signals or the γ- capro lactones in the environment can lead to the transcription of qsdA within qsd operon (Barbey et al., 2012, 2013). A putative transcriptional regulator homologous to TetR (QsdR) had been reported upstream of *qsd* operon (Latour et al., 2013), which, in the absence of AHL could bind to the promoter inhibiting the expression of qsdA. In the presence of AHL or γ-butyro lactones, the QsdR might undergo conformational change leading to the transcription of the gene qsdA (Cuthbertson and Nodwell, 2013; Barbey et al., 2018).

Attenuation of QS-regulated pathogenesis in *Pectobacterium carotovorum subsp. carotovorum*, a pathogen of *Solanum tuberosum* (potato tubers), by rhizosphere soil *Rhodococcus erythropolis* W2 illustrates the interaction between a QS producer, a QQ producer, and their plant host. The treatment of rhizosphere soil of potato plant with growth stimulator such as gamma- caprolactone (GCL), provoked the growth of native AHL degrading strains especially *Rhodococcus erythropolis* (Cirou et al., 2007, 2011). Another study with *Rhodococcus* sp. R138 isolated from GCL treated potato rhizosphere soil exhibited strong biocontrol activity in potato tuber assay by degrading AHL and through assimilating GCL (Cirou et al., 2011). *Rhodococcus erythropolis* not only increased its population in response to GCL (a natural plant molecule) but also assimilated GCL, a reaction proposed to have been catalyzed by QsdA and other rhodococcal enzymes (Cirou et al., 2012). Drastic reduction in AHL mediated virulence of *Pectobacterium atrospeticum by Rhodococcus erythropolis* was identified by transcriptome analysis (Kwasiborski et al., 2015). *Rhodococcus* sp. BH4 encapsulated within free moving alginate cell trapping beads (CEBs) quenched AHL and reduced the synthesis of extracellular matrix of biofilm-forming microbial cells in membrane bioreactors. This property of quenching AHL by strain BH4, in combination with the physical friction exerted by alginate beads, has been proposed as prospective model for controlling biofouling (Kim et al., 2013).

## Family: *Streptomycetaceae*; Genus: *Streptomyces*

An AHL acylase termed as AhlM (N-acyl homoserine lactone acylase) derived from *Streptomyces* sp. strain M664 was the first AHL degrading enzyme characterized from the genera *Streptomyces* (Park et al., 2005). Discovered based on its potential to obstruct N-acyl homoserine lactone facilitated violacein production, the AHL acylase catalyzed the hydrolysis of an amide bond between homoserine lactone and acyl side chain in AHL. The active enzyme was composed of 804 amino acids that were arranged in a pattern characteristic of a penicillin acylase class of proteins belonging to Ntn hydrolase superfamily. Amino acid sequence analysis of AhlM with known AHL acylases: AiiD from *Ralstonia* strain XJ12B (Lin et al., 2003) and PvdQ from *Pseudomonas aeruginosa* (Huang et al., 2003) displayed < 35% sequence identity. Apart from the acylase activity, the AhlM also displayed deacylation activity against long acyl chain AHLs and was suggested of possessing the ability to degrade cyclic lipopeptides. At a concentration of 20 μg/ml, AhlM significantly reduced the production of elastase, total protease, and Las A protease in *P. aeruginosa* PAO1 (Park et al., 2005).

A metabolite phenylalanyl-ureido-citrullinyl-valinyl-cycloarginal termed as FA-70C1 **(4)** (Tables 1, 3) isolated from *Streptomyces* species FA-70, strongly inhibited arg-gingipain (Rgp), an enzyme crucial for survival and proliferation of *Porphyromonas gingivalis* both *in vitro and in vivo* (Kadowaki et al., 1998, 2003).

Guadinomines A **(5)** and B **(6)** (Table 3) derived from *Streptomyces* K01-0509 showed dose-dependent inhibitory activity against hemolysis caused by enteropathogenic *Escherichia coli* (EPEC), potentially through the inhibition of type III secretion system. The inhibitory concentration (IC_50_) value of guadinomine B and guadinomine A was 0.007 mg/ml and 0.02 mg/ml, respectively (Iwatsuki et al., 2008). Piericidin A1 **(7)**, a major metabolite of *Streptomyces* sp. TOHO-Y209 and TOHO-O348, displayed an IC_50_ value of 10 μg/ml against violacein production by *C. violaceum* CV026. 3′-rhamnopiericidin A1 **(8)**, and piericidin E **(9)** also expressed QSI activity but much lesser than piericidin A1 (Ooka et al., 2013). Alnumycin D **(10)**, a C-ribosylated pathway shunt product isolated from recombinant strain *Streptomyces albus*, effectively inhibited the biofilm and planktonic cells of *Staphylococcus aureus* ATCC 25923 by 12 to 22-fold higher than alnumycin A. Similarly, granaticin B, a polyketide metabolite from *Streptomyces violaceoruber*, could disrupt pre-formed staphylococcal biofilms. The structural similarities observed between the two compounds, including glycosylation at the C-8 position with ribopyranosyl unit in alnumycin D and the aglycone unit through C–C bond at C-7 and C-8 positions in granaticin B, were suggested to have contributed to the biofilm inhibitory activity. In addition to this, the oxygenation pattern within the naphthoquinone ring, carbonyl oxygen atom in alnumycin D and hydroxyl group in granaticin B, were also suggested to have contributed to the anti-biofilm activity (Oja et al., 2015).

Well studied for its role in suppressing (Tzaridis et al., 2016) and treating tumors (Walsh et al., 2016; Das et al., 2017; Schmidt et al., 2017; Lamture et al., 2018), actinomycin D from *Streptomyces parvulus* also possessed biofilm inhibitory activity *in vitro*. At 0.1 μg/ml concentration, actinomycin D reduced the formation of biofilm of methicillin sensitive *Staphylococcus aureus* strains (ATCC 25923 and ATCC 6538) and methicillin resistant *Staphylococcus aureus* strain (ATCC 33591) by ≥ 70%, ≥ 80%, and ≥ 80%, respectively (Lee et al., 2016). At the same concentration, actinomycin D reduced the biomass and mean thickness of *Staphylococcus aureus* biofilm by 98%, and the hemolytic activity by ≥ 85%. This led to the suggestion that the inhibitory activity of actinomycin D toward *Staphylococcus aureus* was partly concatenated with its ability to inhibit hemolysis. Besides, *Streptomyces parvulus* derived actinomycin D also reduced the hydrophobicity of the staphylococcal cells, a property crucial for the bacterial adherence to the substrata (Krasowska and Sigler, 2014). The failure of the actinomycin D to disperse preformed staphylococcal biofilms highlighted the non-association of actinomycin D with protease or the staphylococcal agr QS system (Lee et al., 2016). Conversely, actinomycin D derived from *Streptomyces parvulus* HY026 significantly reduced the production of violacein by *C. violaceum* up to 90.7% at 50 μg/ml concentration. Although the potential of actinomycin D from endophytic *Streptomyces parvulus* (1% (v/v) concentration) to inhibit staphylococcal biofilms does seem to be more superior than the actinomycin D from *Streptomyces parvulus* HY026 (10% v/v concentration), the non-agr QS mediated mode of biofilm inhibition by the former strain and anti-QS activity of actinomycin D from HY026 exemplifies the outstanding functional adaptation of actinomycin D at molecular level (Miao et al., 2017; Table 1).

*Streptomyces coelicoflavus* S17 derived 1H-pyrrole-2-carboxylic acid **(11)** and docosanoic acid **(12)** (Table 3) significantly attenuated the virulence of *P. aeruginosa* PAO1 at 1 mg/ml concentration. While 1H-pyrrole-2-carboxylic acid decreased the production of elastase, protease, and pyocyanin by 96, 74, and 44%, respectively, the docosanoic acid reduced their production by 91.8, 46.1, and 64.45%, respectively. The compound 1H-pyrrole-2-carboxylic acid eliminated the expression of las genes; lasA, lasB, lasI and lasR by 88, 92, 80, and 87%, respectively. The compound also inhibited rhl/pqs cascade including pqsA, pqsR, rhlI and rhlR by 97, 78, 69, and 89%, respectively (Hassan et al., 2016). All maniwamycins from *Streptomyces* TOHO-M025 reduced the production of violacein by *C. violaceum* CV026 in a dose-dependent manner at a concentration ranging from 0.01 to 1 mg/ml. Maniwamycins D **(13)** and E **(14)** displayed higher QS inhibitory activity than C **(15)** and F. Maniwamycin E showed IC_50_ value of 0.12 mg/ml (Fukumoto et al., 2016).

Quercetin **(16)** from marine *Streptomyces fradiae* PE7 reduced the germination of *Anabaena* and *Nostoc* sp. spores by 70% at 100 μg/ml concentration (Gopikrishnan et al., 2016). The addition of culture extract from *Streptomyces xanthocidicus* KPP01532 (≥ 2.5 μL), reduced the violacein production by CV026 considerably. Transcriptomic analysis on the effect of purified piericidin A **(17)** and glucopiericidin A **(18)** from the KPP01532 media extract on *E. carotovora subsp. atroseptica* revealed that the reduction in the expression of genes encoding hydrolytic enzymes including pectate lyase (*PelC*), cellulase *(CelV*), polygalacturonase (*PehA*) and QS controlled virulence-associated gene (*nip*). Treatment of potato tubers with 50 and 100 μM of piericidin A also reduced the development of soft rot disease symptoms. Similar results were also obtained *in vitro* with KPP01532 glucopiericidin A (Kang et al., 2016).

Hygrocin C (an ansamycin) derived from *Streptomyces* sp. SCSGAA0027 displayed a biofilm inhibitory concentration (BIC_80_) value of 12.5 μg/ml, 25.0 μg/ml and 200 μg/ml against *Bacillus amyloliquefaciens, Staphylococcus aureus* and *P. aeruginosa*, respectively. At a dosage of 12.5 to 100 μg/ml, hygrocin C reduced pre-formed biofilms of *Bacillus amyloliquefaciens* by 11.73 to 54.76%. Transcriptomic analysis showed that in the presence of hygrocin C, 107 genes were upregulated, and 102 genes were downregulated. While the downregulated genes were crucial for motility including FliC and FliA (Flagellar genes), MotB (Flagellar motor protein) and two-component systems including ResE (Sensor histidine kinase ResE) and CydB (Cytochrome-bd-ubiquinol oxidase), the upregulated genes led to the mass synthesis of arginine and histidine. The unbalanced level of histidine and arginine, and the downregulation of genes essential for motility were suggested to have contributed to the repression of biofilm formation. It was also suggested that the suppression of bacteria’s survival was due to the downregulation of nitric oxide dioxygenase (HmpA) (Wang et al., 2018).

Metal nanoparticles including selenium and silver nanoparticles synthesized from *Streptomyces* species have also been effective in attenuating virulence of microbial pathogens. Selenium nanoparticles synthesized by *Streptomyces minutiscleroticus* M10A62 inhibited biofilm of antibiotic-resistant strains of *Acinetobacter* species at a concentration of 3.2 μg/ml (Ramya et al., 2015). Silver nanoparticles from *Streptomyces griseorubens* AU2 suppressed the biofilm of *Staphylococcus aureus* ATCC 25923 and *P. aeruginosa* ATCC 27853 at a concentration 20 μg/ml and 10 μg/ml, respectively (Baygar and Ugur, 2017). A furonone derivative from *Streptomyces* sp. AT37 5-[(5E,7E,11E)-2,10-dihydroxy-9,11-dimethyl-5,7,11-tridecatrien-1-yl]-2-hydroxy-2-(1-hydro-xyethyl)-4-me- thyl-3(2H)-furanone or antibiotic AT37-1 **(19)** exhibited minimum biofilm inhibition concentration (MBIC_50_) of 10–15 μg/mL against methicillin ensitive *Staphylococcus aureus* (MSSA) ATCC 29523 and methicillin resistant *Staphylococcus aureus* (MRSA) ATCC 43300 (Driche et al., 2017). Streptorubin B from *Streptomyces* sp. strain MC11024 displayed IC_50_ value of 0.56 μM against the biofilms of m MRSA N315. Although streptorubin B inhibited the growth of MRSA N315 at 2–4 μg/mL, the compound also exhibited anti-biofilm activity (Bauermeister et al., 2019).

At a dosage of 2.5% (v/v), the metabolites from marine *Streptomyces albus* A66 repressed the formation of *V. harveyi* biofilms by 99.3% and dispersed the mature biofilms of *V. harveyi* by 75.6%. The A66 metabolite was suggested to affect the development of *Vibrio* biofilms by attenuating the initiation and maturation stage (You et al., 2007; Table 2). Methanolic extract from the spent medium of *Streptomyces akiyoshiensis* CAA-3 inhibited staphylococcal biofilms at a concentration of 0.1 mg/ml. The extract also possessed the ability to inhibit the colonization of *Staphylococcus aureus* in the intestine of *Caenorhabditis elegans* up to 70% (Table 2; Bakkiyaraj and Pandian, 2010). Culture extracts of *Streptomyces* sp. BFI 250 at 0.01% (v/v) inhibited the biofilm formation and detachment of preformed biofilms of *Staphylococcus aureus* ATCC 25923 by ≥ 80% for more than 17 h. The ability to subdue both the formation and detachment of biofilms by *Streptomyces* sp. BFI 250 was due to the extracellular protease in the extract that was equivalent to approximately 0.1 μg of proteinase K/ml (Park et al., 2012). Extracts from *Streptomyces* sp. NIO 10068 spent medium reduced motility, formation of biofilm, production of pyocyanin, rhamnolipid and Las A protease, swimming and twitching by 90, 67, 45, 45, 43, 20, and 15%, respectively in *P. aeruginosa* ATCC 27853. Among the several active compounds including cinnamic acid, linear dipeptides N-amido-a-proline, pro-line–glycine and aromatic acids characterized from the extract of strain NIO 10068, only linear dipeptide and cinnamic acid expressed quorum sensing inhibitory (QSI) activity (Naik et al., 2013). DNA microarray analysis revealed that the spent medium of the strain BFI 230 repressed 42 genes and induced 78 genes in *P. aeruginosa* cells embedded within the biofilm. The 78 genes that were induced were essential for utilization of iron, biosynthesis of phenazine (phz operon), pyoverdine (pvd operon) and pyochelin (pch). At 1% (v/v) concentration, spent medium of BFI 230 repressed 90% of the *P. aeruginosa* biofilm. However, at this concentration other virulence factors including swarming and the production of pyoverdine and pyocyanin increased. As the transcriptomic analysis showed that the BFI 230 spent medium induced the genes for iron uptake, external addition of ferrous compounds (FeCl_3_ and FeSO_4_) in the presence of the BFI 230 spent medium resulted in the restoration *P. aeruginosa* biofilms. The study revealed that proteins or peptides native to the *Streptomyces* sp. BFI 230 spent medium suppressed the formation of *P. aeruginosa* biofilms either indirectly interfering with the bacterium’s iron utilization or through linking iron with quorum sensing system (Kim et al., 2012).

Characterization of quorum quenching activity in 63 *Streptomyces* soil isolates showed that 3 strains St11, St61 and St62 degraded synthetic hexanoyl homoserine lactone (HHL). The acylase was stable in the presence of heavy metals and chelating agents, and maintained a maximum catalytic activity between 20 to 50°C up to pH 8 (Sakr et al., 2015). The extracts of *Streptomyces akiyoshinensis* (A3) inhibited *Streptococcus pyogenes* biofilms at a concentration of 10 to 50 μg/ml. The extract from *Streptomyces akiyoshinensis* affected the cell hydrophobicity, and the initial colonization of *Streptococcus pyogenes* (Nithyanand et al., 2010). About 200 μg/ml of diethyl ether extracts of *Streptomycetes* species A745 culture subdued the formation of *V. cholerae* biofilm by 60% (Augustine et al., 2012). Crude fatty acid extract from three *Streptomyces* isolates (*Streptomyces sps* isolates S8, S9, and S15) inhibited formation of *Streptococcus pyogenes* ATCC 19615 biofilm at a concentration of 10 μg/ml. Remarkably, the lipids found in the crude extract of these *Streptomyces* species influenced the secretion of extracellular proteins especially streptolysin S (Rajalakshmi et al., 2014). The extract of *Streptomyces* sp. SBT343 displayed BIC_50_ value of 62.5 μg/ml toward *Staphylococcus epidermidis* RP62A biofilm. At 125 μg/ml, the extract subdued the formation of biofilms of MRSA, MSSA and *Staphylococcus epidermidis*. Physiochemical characterization of the extract revealed that the bioactive molecule(s) mediating the inhibitory activity toward staphylococcal biofilm were thermostable and non-proteinaceous in nature (Balasubramanian et al., 2017). Hexane partition of *Streptomyces* sp. CCB-PSK207 spent medium gradually increased the survival of *P. aeruginosa* PA14 infected *C. elegans* from 45.33 to 72.71% at the concentration ranging from 50 to 400 μg/ml. Phenotypical analysis on the expression of virulence factors of PA14 showed that the metabolites (fatty acid methyl esters) in the extract were indifferent on the formation of biofilm or on the production of protease and pyocyanin. However, restoration of the green fluorescent protein (GFP) expression in transgenic lys-7:GFP *C. elegans* strain SAL105 revealed that the hexane partition of CCB-PSK207 did not repress the killing of *C. elegans* by subduing the virulence of PA14, but rather through boosting the immunity in the nematode by inducing the expression of lysozyme 7 (*lys*-7) (Fatin et al., 2017). The minimum biofilm inhibitory concentration of metabolites from *Streptomyces albogriseolus* GIS39Ama were 312 ppm against *Escherichia coli* MTCC 687, 625ppm against *Klebsiella pneumoniae* MTCC 3384 and *Vibrio cholerae* MTCC 3906, and 1250 ppm against *Pseudomonas aeruginosa* MTCC 2453. *Streptomyces albogriseolus* GIS39Ama reduced the production of violacein by *C. violaceum* MTCC 2656 by 87.67% (Lokegaonkar and Nabar, 2017). The extract from *Streptomyces* sp. MC025 isolated from an unidentified red alga suppressed the formation of *Staphylococcus aureus* biofilm by ≥ 90% with minimal bactericidal effect on planktonic cells. Bioactivity-guided fractionation of the crude extract *Streptomyces* sp. MC025 led to the identification of 6 bipyridines molecules, of which, collismycins C **(20)** and pyrisulfoxin A **(21)** showed inhibitory activity against MSSA ATCC 6538 at 50 μg/mL. Further studies revealed that Collismycin C was the major component initiating anti-biofilm activity by chelating Fe ions, and that the location of the OH group on bipyridines were vital for anti-biofilm activity against *Staphylococcus aureus* (Lee et al., 2017).

Screening of 101 marine *Actinomycetes* led to the discovery of *Streptomyces* strains that could suppress biofilms of *Escherichia coli* (by 61 – 80%) and *Staphylococcus aureus* (by 60%) (Leetanasaksakul and Thamchaipenet, 2018). Extracts from the spent medium of *Streptomyces* sp. TRM 41337 suppressed the formation of *Staphylococcus epidermidis* (ATCC 35984 and 5-121-2) biofilms by ≥ 90% in a dose-dependent manner for over 24 h. While the culture extracts of *Streptomyces* sp. TRM 41337 effectively degraded DNA of *S. epidermidis*, the protein metabolite from the extract reduced the cell surface hydrophobicity and degraded EPS of *Staphylococcus epidermidis*. Thus, it was suggested that through these properties, the crude protein was able to prevent the formation of *S. epidermidis* biofilm (Xie et al., 2018).

Melanin pigment (soluble and insoluble forms) purified from *Streptomyces* sp. ZL-24 suppressed the formation of *P. aeruginosa* ATCC 9027 and *Staphylococcus aureus* ATCC 6538 biofilms up to 67.5 and 74.6% and 79.2 and 71.7%, respectively (Wang et al., 2019). Similarly, the extract from *Streptomyces griseoincarnatus* HK12 suppressed *P. aeruginosa* and *Staphylococcus aureus* biofilms by 82.657 and 78.973%, respectively. GC-MS analysis of the extract showed the presence of five active compounds including arachidic acid, erucic acid, 13Z-octadecenal **(22)**, 9Z-octadecenal and tetracosanoic acid. *In silico* docking of all the five active compounds with LasI of *P. aeruginosa* showed that the 13Z-octadecenal interacted with LasI and formed pi-alkyl bond with the conserved residues Trp33 and Phe2 in LasI. It was suggested that the downregulation of QS-regulated virulence gene was due to the small molecule mediated inhibition of LasI binding to its native ligand LasR. This study also suggested that the presence of fatty acyl molecule in HK12 spent medium could have exerted both synergistic or independent anti-biofilm activity (Kamarudheen and Rao, 2019). Four new α- pyridones (compound 16 **(23)**,17 **(24)**,19 **(25)** & 20 **(26)**) generated through chemical transformation of the compounds derived from culture extract of *Streptomyces* sp., inhibited the expression of *P. aeruginosa* QSIS-lasI biosensors at a concentration of 6.35 μg/well (Du et al., 2018).

Dimorphic fungi *Candida albicans* can potentiate clinically significant systemic infections due to its complex and multifactorial virulence factors including isocitrate lyase (ICL), a glyoxylate cycle enzyme (Lorenz and Fink, 2001; Ramírez and Lorenz, 2007; Mayer et al., 2013). Bahamaolide A **(27)** purified from *Streptomyces* sp. *CNQ343* strongly inhibited the mRNA expression of ICL with an IC_50_ value of 11.82 μM. Due to the absence of ICL in mammals, Bahamaolide A has been suggested as a promising anti-virulent agent for *C. albicans* (Lee et al., 2014).

Pre-exposure of *C. albicans* to the *Streptomyces toxytricini* Fz94 culture extract at a concentration of 5 g/L prevented the formation of biofilm up to 92%. At 7 g/L, the extract destroyed up to 82% of biofilms after 120 min (Sheir et al., 2017). Partially purified fractions of *Streptomyces chrestomyceticus* strain ADP4 strongly inhibited the secretory aspartic proteases (Saps) in *C. albicans* which has been shown to be vital for the formation of hyphae, phenotypic switching, adhesion, digestion of host cell membrane, and also for the evasion of host immune system by the yeast (Srivastava et al., 2017). A metabolite from *Streptomyces* sp. ADR1 displayed MBIC ≤ 15.625 μg/ml and < 500 μg/ml against preformed biofilm of pathogenic *Staphylococcus aureus* (Singh and Dubey, 2018). Khatmiamycin **(28)** and aloesaponarin II **(29)** derived from *Streptomyces* sp. ANK313 inhibited the motility of zoospores of *Plasmopara viticola* with a MIC value of 10 μg/ml and 25 10 μg/ml, respectively (Abdalla et al., 2011).

## Others

Partially purified pigment from *Actinomycetes* C5-5Y inhibited the cell surface hydrophobicity, proteolytic and lipase activity of *Streptococcus mutans* and *Staphylococcus aureus* (Table 2). When treated with the pigment, cell surface hydrophobicity of these nosocomial pathogens reduced by 23 and 24% compared to the 91 and 89% hydrophobicity observed in the control cells. Furthermore, at 10 μg/ml concentration, the pigment also significantly reduced the formation of *Streptococcus mutans* and *Staphylococcus aureus* biofilms, leading to the suggestion that *Actinomycetes* C5-5Y derived pigment were capable of quenching quorum sensing signals (Soundari et al., 2014). Transcriptomic analysis on the effect of cyclodepsipeptides (WS9326A and WS92326B) from *Actinomycetes* strain DSW812 on the VirSR system of *C. perfringens*, revealed that the WS9326A suppressed the expression of pfoA encoding perfringolysin O in dose-dependent manner at sub-micromolar IC_50_ concentration. As WS9326B lacked this activity, the absence of double bonds in the dehydrotyrosine of WS92326B was concluded to be crucial for the cyclodepsipeptide binding to VirS system. However, the study also showed that WS9326B effectively decreased the cytotoxicity of *Staphylococcus aureus* on human corneal epithelial cells significantly. WS9326A and WS9326B also repressed hemolysin production by *S. aureus* 8325–4 (type-I AIP), *S. aureus* K12 (type-II AIP) and *S. aureus* K9 (type-IV AIP), indicating the specificity of *Actinomycetes* cyclodepsipeptides toward the different auto inducing peptides (AIP). Cochinmicins II and III from *Actinomycetes* strains GMKU369, have also been suggested to function as an antagonist like cyclodepsipeptides due to their similarities in structure, molecular size, and hydrophobicity (Desouky et al., 2015).

## Opinion and Future Perspective

**FIGURE 1:**
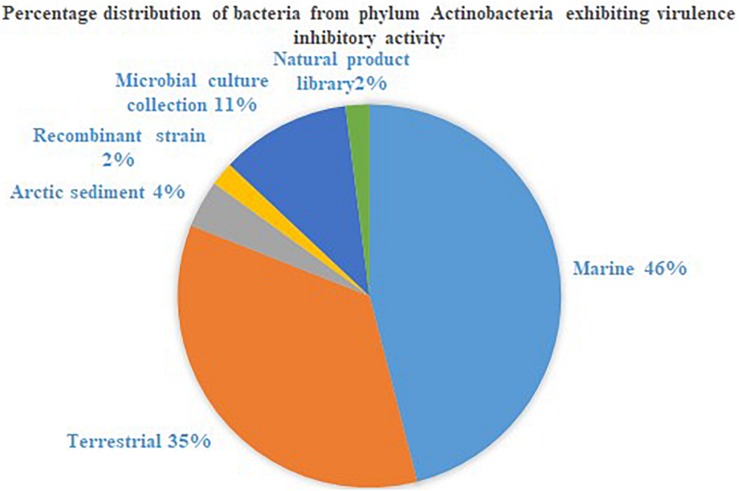
Percentage distribution of bacteria from phylum Actinobacteria exhibiting virulence inhibitory activity.

**FIGURE 2:**
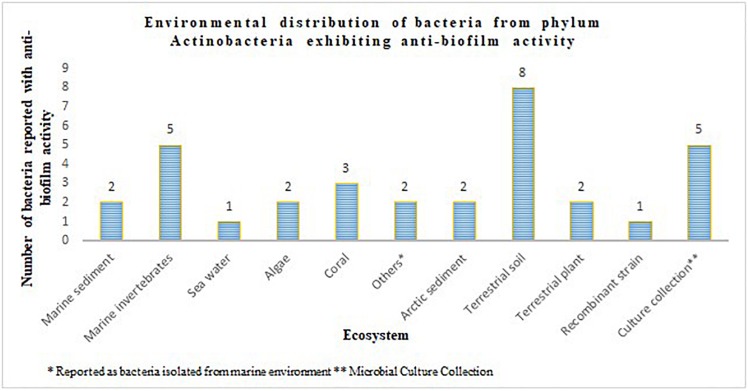
Environmental distribution of bacteria from phylum Actinobacteria exhibiting anti-biofilm activity.

The phylum Actinobacteria encompasses a group of organisms well known for its prodigious production of secondary metabolites with complex scaffolding and chemical entities. This actinic uniqueness has been beneficial in terms of its pharmaceutical adaptability, as clinically significant antimicrobials, anti-tumor agents, immunosuppressants, anti-proliferative agents, anti-parasitic agents and herbicides than any other bacterial origin natural product. In this regard, identification of secondary metabolites from the phylum Actinobacteria with potential to attenuate virulence in other microorganisms, and the broad-spectrum specificity toward different AHLs, could be advantages for engineering the much anticipated anti-virulence drugs. Actinobacteria strains that suppressed microbial virulence have been reported majorly from marine and terrestrial environment (Figures 1, 2). Over the past decade, several marine natural products (MNP) derived from various phyla of bacteria, alga, seaweeds and invertebrates exhibiting anti-virulence property including anti-biofilm property have been reported. This could be the reflection of the recent trend in exploring the metabolite profile of microbiome from uninhabited areas including arctic regions, to prevent the re-isolation of known active metabolites. While the active metabolites from the Actinobacteria have been demonstrated with virulence suppressing potential against a wide range of bacteria and yeast cells, the assays employed to evaluate the virulence inhibiting potential are very limited (Table 4). The Actinobacteria derived products were mainly evaluated for their potential to inhibit biofilm formation or the production of enzymes, pigments, cell hydrophobicity, and motility. Yet, many crucial virulence factors including iron uptake, immune cell evasion and suppression of host immune system should have been considered as promotion of pathogenesis by bacteria like *Staphylococcus aureus* is site-specific. Similarly, evaluation of the majority of actinobacterial origin anti-virulence agents has been against very limited bacterial reference strains and reporter strains particularly *Staphylococcus aureus* and *Pseudomonas aeruginosa.* Although, undeniably, these organisms are highly virulent with or without AMR, researches with a wide range of organisms especially variant cell populations such as persister cells that have been demonstrated to be the etiological agents of chronic infections would help to establish the potency of metabolites as anti-virulences. To conclude, in the evolutionary struggle for co-existence between microorganism and humans, the single-sided supremacy observed during the prodromal antibiotic era convincingly advocates requirement of multifactor approach to target pathogenesis of microorganism in the host body.

**TABLE 4:** List of compounds characterized from Actinobacteria and their specific virulence inhibitory function.

**Target Organisms**	**Virulence determinants evaluated**	**Actinobacterial compound exhibiting anti-virulence property**
*P. aeruginosa* (ATCC 27853, ATCC 10145, ATCC 9027, *PA01*)	Motility (Swimming and Twitching)	Cinnamic acid linear dipeptide (Pro-Gly and N-amido-α-proline)
	Biofilm	B4-EPS1 Actinomycin D Cinnamic acid linear dipeptide (Pro-Gly and N-amido-α-proline) Melanin 9Z-Octadecenal Arachidic acid, Erucic acid 13Z-Octadecenal and tetracosanoic acid, Silver nano particles
	Pyocyanin	Cinnamic acid linear dipeptide (Pro-Gly and N-amido-α-proline) 1H-pyrrole-2-carboxylic acid Docosanoic acid
	Rhamnolipid	Cinnamic acid linear dipeptide (Pro-Gly and N-amido-α-proline)
	Production of elastase	1H-pyrrole-2-carboxylic acid Docosanoic acid
	Production of protease	1H-pyrrole-2-carboxylic acid Docosanoic acid
	Expression of las genes	1H-pyrrole-2-carboxylic acid 4-Hydroxy-3-methyl-6-propylpyridin-2(1H)-one 3-Ethyl-4-hydroxy-6-isopropylpyridin-2(1H)-one 4-Hydroxy-6-isobutyl-3-methylpyridin-2(1H)-one (S)-6-(sec-Butyl)-4-hydroxy-3-methylpyridin-2(1H)-one Nocapyrone H Nocapyrone I Nocapyrone M
	Expression of rhl/pqs cascade	1H-pyrrole-2-carboxylic acid
*Staphylococcus aureus* (ATCC 25923, ATCC 6538, ATCC 33591, ATCC 95005, N315)	Biofilm	Protease Actinomycin D Alnumycin D Granaticin B Streptorubin B Melanin 5-[(5E,7E,11E)-2,10-dihydroxy-9,11-dimethyl-5,7,11-tridecatrien-1-yl]-2-hydroxy-2-(1-hydro-xyethyl)-4-me- thyl-3(2H)-furanone Collismycin C Silver nano particles
	Hemolysis	Actinomycin D WS9326A WS9326B
	Slime	Actinomycin D
	Cell hydrophobicity	Actinomycin D
*Staphylococcus aureus* agr reporter strain 8325-4	Agr-dependent gene expression	Arthroamide Turnagainolide A

## Author Contributions

Both authors contributed equally to the preparation and completion of the manuscript.

## Conflict of Interest Statement

The authors declare that the research was conducted in the absence of any commercial or financial relationships that could be construed as a potential conflict of interest.

## Footnotes

1 http://www.bacterio.net
